# Revisiting QRS Detection Methodologies for Portable, Wearable, Battery-Operated, and Wireless ECG Systems

**DOI:** 10.1371/journal.pone.0084018

**Published:** 2014-01-07

**Authors:** Mohamed Elgendi, Björn Eskofier, Socrates Dokos, Derek Abbott

**Affiliations:** 1 Department of Computing Science, University of Alberta, Edmonton, Alberta, Canada; 2 Pattern Recognition Lab, Friedrich-Alexander-University Erlangen-Nuremberg, Bavaria, Germany; 3 Graduate School of Biomedical Engineering, University of New South Wales, Sydney, New South Wales, Australia; 4 School of Electrical and Electronic Engineering, University of Adelaide, Adelaide, South Australia, Australia; Northwestern University, United States of America

## Abstract

Cardiovascular diseases are the number one cause of death worldwide. Currently, portable battery-operated systems such as mobile phones with wireless ECG sensors have the potential to be used in continuous cardiac function assessment that can be easily integrated into daily life. These portable point-of-care diagnostic systems can therefore help unveil and treat cardiovascular diseases. The basis for ECG analysis is a robust detection of the prominent QRS complex, as well as other ECG signal characteristics. However, it is not clear from the literature which ECG analysis algorithms are suited for an implementation on a mobile device. We investigate current QRS detection algorithms based on three assessment criteria: 1) robustness to noise, 2) parameter choice, and 3) numerical efficiency, in order to target a universal fast-robust detector. Furthermore, existing QRS detection algorithms may provide an acceptable solution only on small segments of ECG signals, within a certain amplitude range, or amid particular types of arrhythmia and/or noise. These issues are discussed in the context of a comparison with the most conventional algorithms, followed by future recommendations for developing reliable QRS detection schemes suitable for implementation on battery-operated mobile devices.

## Introduction

According to the World Health Organization, cardiovascular diseases (CVDs) are the number one cause of death worldwide [1]. An estimated 17.3 million people died from CVDs in 2008, representing 30% of all global deaths [1]. Moreover, it is expected that the number of mortalities due to CVDs, mainly from heart disease and stroke, will reach 23.3 million by 2030 and are projected to remain the single leading cause of death for several decades [2].

In 2010, the global direct and indirect cost of CVD was approximately $863 billion and is estimated to rise by 22% to $1,044 billion by 2030. Overall, the cost for CVD alone is projected to be as high as $20 trillion over the next 20 year period [3].

As a consequence of direct and indirect costs of CVD, medical researchers have placed significant importance on cardiac health research. This has led to a strong focus on technological advances with respect to cardiac function assessment. One such research pathway is the improvement of conventional cardiovascular-diagnosis technologies used in hospitals/clinics.

The most common clinical cardiac test is electrocardiogram (ECG) analysis. It represents a useful screening tool for a variety of cardiac abnormalities because it is simple, risk-free, and inexpensive [4]. Advances in technology have led to much change in the way we collect, store and diagnose ECG signals, especially the use of mobile phones to implement the clinical routine of ECG analysis into everyday life [5]–[9]. Thus, in the near future, it is expected that Holter devices, which are traditionally used for ECG analysis in the clinic, will be replaced by portable, battery-operated devices such as mobile phones in the near future [10]. The reason is that Holter devices do not detect arrhythmias automatically in real-time, and do not provide real-time information to the hospital/doctor/patient when a critical heart condition occurs.

Moreover, the advances in memory/storage technology have enabled us to store more ECG signals than ever before. Therefore, researchers are collecting more information in order to understand the mechanisms underlying CVDs, which is expected to ultimately lead to effective treatments. The trend towards using mobile smart phones for ECG assessment further speeds up this process, as the conveniently collected data can potentially be added to databases via the existing internet.

The analysis of ECG signals collected by a mobile phone needs to be fast and feasible in real-time, despite the existing limitations in terms of phone memory and processor capability. The same holds for the ability to analyse large ECG recordings collected over one or more days.

Recently, researchers have put an increased effort into developing efficient ECG analysis algorithms to run within mobile phones, including algorithms for determining the quality of collected ECG signals [11]. This increased effort is also evidenced in the 2011 PhysioNet/Computing in Cardiology Challenge [12], which has been established to encourage the development of ECG software that can run on a mobile phone, recording an ECG and providing useful feedback about its quality.

PhysioNet provided a large set of ECG records for use in their Cardiology Challenge, along with an open-source sample application for an Android phone (Google Inc., USA), and that can classify ECGs as acceptable or unacceptable. Therefore, the next step is to analyse the acceptable ECG signal for diagnosis, without relying on an expert for interpretation. If this possibility becomes a reality, it will help developing nations and rural populations, by benefitting from otherwise inaccessible expertise.

**Figure 1:**
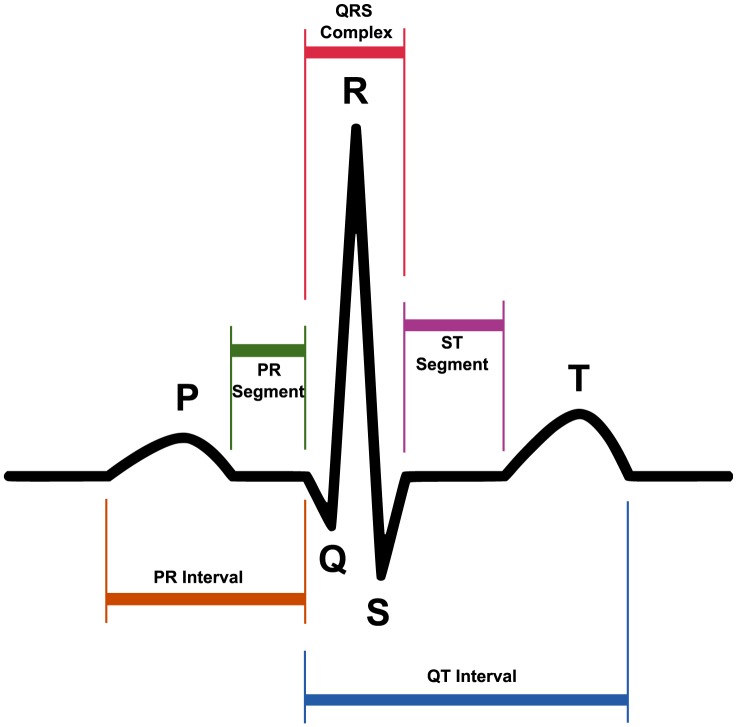
Main Events in ECG signals. A typical ECG trace of the cardiac cycle (heartbeat) consists of a P wave, a QRS complex, and a T wave.

Note that ECG signals contain features that reflect the underlying operation of the heart. These features represent electrophysiological events that coincide with the sequence of depolarisation and repolarisation of the atria and ventricles. The signal of each heartbeat contains three main events: the P wave, the QRS complex, and the T wave (as shown in Figure 1). Each event (wave) has its corresponding peak. The analysis of ECG signals for monitoring or diagnosis requires the detection of these events. Once an event has been detected, the corresponding signal can be extracted and analysed in terms of its amplitude (peak), morphology, energy and entropy distribution, frequency content, intervals between events and other more complex parameters. The automatic detection of the P, QRS and T events is critical for reliable cardiovascular assessment, such as diagnosing cardiac arrhythmias [13]–[17], understanding autonomic regulation of the cardiovascular system during sleep and hypertension [18], [19], detecting breathing disorders such as obstructive sleep apnea syndrome [20], [21], and monitoring other structural or functional cardiac disorders. Once the QRS, P and T events are detected accurately, a more detailed analysis of ECG signals can be performed.

The detection of QRS complexes has been extensively investigated over the past two decades. Many attempts have been made to find a satisfying universal solution for QRS complex detection. Difficulties arise mainly because of the diversity of the QRS waveforms, abnormalities, low signal-to-noise ratio (SNR) and as well as artefacts accompanying ECG signals. Conversely, P and T event detection has not been investigated as much as QRS detection, and the P and T event detection problem is still far from being solved [22]. Reliable P and T wave detection is more difficult than QRS complex detection for several reasons, including low amplitudes, low SNR, amplitude and morphology variability, and possible overlapping of the P wave and the T wave. Any cardiac dysfunction associated with excitation from ectopic centres in the myocardium may lead to premature complexes (atrial or ventricular), which change the morphology of the waveform and the duration of the RR interval. The occurrence of multiple premature complexes is considered clinically important, as it indicates disorders in the depolarisation process preceding the critical cardiac arrhythmia. For all the above-mentioned reasons, the accurate detection of QRS complexes is clinically important. Prior to developing a fast-robust QRS detector that suits battery-driven applications and continuous 24/7 ECG monitoring, it is necessary to evaluate the performance of the current algorithms against the following three assessment criteria:

1. **Robustness to noise**: there are several sources of noise (e.g. power line interference, muscle noise and motion artefacts). Therefore, the developed algorithms should be robust to these noise sources.
2. **Parameter choice**: The choice of parameters must lead to accurate detection. Parameters must not have to be manually adjusted for different recordings.
3. **Numerical efficiency**: The developed algorithm may have a large number of iterations, parameters to adjust, features extracted, or classification steps. It is desirable to provide numerically efficient (simple, fast, and fewer calculations) algorithms. Of course, computers have become very fast, and therefore numerical efficiency is less important than it used to be. However, if a simple and fast algorithm can achieve good results, there is no need for more complex algorithms. In particular, when the algorithm is used online (in a slightly modified form from the offline version) in a mobile phone embedded system, numerical efficiency is still relevant.

In the remainder of this review article, these proposed assessment criteria will be used to evaluate several well-known QRS algorithms in two important stages: QRS enhancement and QRS detection. The QRS enhancement stage is used to enlarge the QRS complex relative to the other ECG features (P, T, and noise). This stage is occasionally referred to as pre-processing or feature extraction. The QRS detection stage is used to demarcate the QRS complex by providing the onset and offset points of the QRS complex, and especially the location of the prominent R peak. The remainder of this paper is structured as follows: the next section delineates several types of QRS enhancements techniques, whilst Section 3 compares different QRS detection methods. Finally a discussion and concluding remarks are presented in Section 4.

In describing the algorithms for QRS enhancement and detection in this article, note that 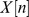 refers to the raw ECG signal collected from any ECG monitoring system, including battery-operated devices; while 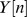 refers to the filtered 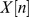 signal.

## QRS Enhancement

**Figure 2:**
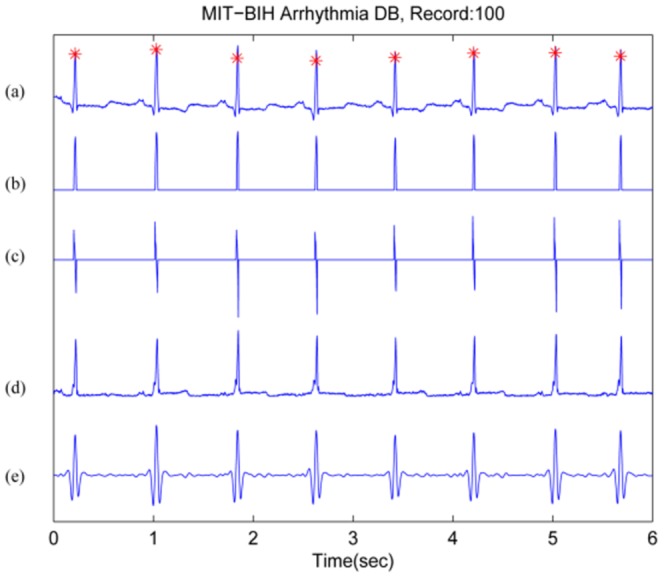
QRS enhancement stage in ECG signals. (a) ECG signal (top: from record 100 of the MIT-BIH Arrhythmia Database [62]), (b) amplitude from Eq.1 where 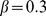, (c) first derivative from Eq.4, (d) first derivative and second derivative from Eq.7, and (e) digital filter from Ref. [33]. Signal amplitudes have been manipulated to fit all signals in one figure. Here, a red asterisk represents the annotated R peak.

This section presents several signal processing techniques [23]–[26] that have been used to emphasise the QRS segment in time, frequency and time-frequency series, as shown in Table 1. Figure 2 demonstrates the importance of the QRS enhancement stage as a prerequisite for detecting the QRS complex.

**Table 1:** Comparison of QRS enhancement techniques based on algorithm usage and assessment criteria.

Technique	Algorithm	Robustness to noise	Parameter choice	Numerical efficiency
*Amplitude*	Amplitude threshold is applied to the ECG signal, usually followed by the first derivative of the ECG signal [23], [24] with a second threshold.	The signal noise is not removed properly and is not considered by the first- derivative-only class of algorithms for feature extraction.	The processed segments have equally fixed lengths [23], [24], [25], [26], [27], [28].The value of the β ratio must be adjusted once before ECG signal analysis takes place. The threshold remains fixed throughout the entire ECG signal analysis [23], [24], [25], [26], [27], [28].Investigators have introduced several differentiators without noting the reason behind their choices [23], [24], [25], [26], [27], [28].The length of the processed ECG segment is determined experimentally [23], [24], [25], [26], [27], [28].Friesen et al. [29] used ECG data with a fixed length of 33 seconds. Their algorithm scored a high accuracy because they processed small segments. It is expected that the performance of this algorithm on longer ECG trace will be poor unless the long ECG signals are separated into smaller segments. In this case, the performance will likely improve, however there is a possibility of losing beats at the beginning and end of each processed ECG segment	Amplitude and first derivative class of algorithms is simple and usually contain a threshold and first derivative equation for feature extraction. The complexity mainly depends on the threshold used and segmentation if applied.
*First Derivative Only*	First derivative of ECG signal followed by threshold [30]–[32]. (thresholding will be discussed in Section 3)Amplitude threshold applied to ECG signal followed by first derivative of ECG signal [23], [24] (see Section 2.2), followed by another thresholdFirst derivative combined with second derivative of ECG signal [26], [27] (see section 2.3), followed by thresholdFirst derivative of ECG signal followed by digital filtering [28] (see section 2.4), followed by thresholdDigital filter applied to ECG signal followed by first derivative [33], followed by thresholdMathematical morphology filtering applied to ECG signal followed by first derivative [34] (see section 2.5), followed by thresholdFirst derivative can be used before applying Hilbert transform [35], [36], [37] (see Section 3.1), followed by thresholdFirst derivative can be used before applying Wavelet transform [38] (see section 4.2), followed by threshold	The first derivative does not remove high-frequency noise; however, it helps to reduce motion artifacts and base line drifts [38].	The processed ECG segments have equally fixed lengths and thresholds [30], [31], [32].As mentioned above, researchers have introduced several differentiators without mentioning the reason behind their choices [30], [31], [32].	First derivative class of algorithms is simple and contains one equation for feature extraction. Most cases used Okada's equation [30]. The complexity of this class will increase if segmentation is applied. The order of complexity depends on the number of processed segments for each record.
*First and Second derivative*	First derivative combined with second derivative of ECG signal [26], [27], followed by threshold.Second derivative can be used before applying Hilbert transform [35], [37] (see Section 3.1), followed by threshold.	The signal noise is not removed properly and is not considered by the first- derivative-only class of algorithms for feature extraction.	The processed segments have equal and fixed lengths [23]–[28].The parameters used are fixed.The choice of the first and second derivative equations is experimentally determined [26], [27]. Moreover, authors do not justify their combination of first and second derivatives.As mentioned above, investigators have introduced various differentiators without noting the reason behind their choices [26], [27].	First- and second-derivative classes of algorithms are simple and contain only up to four equations for feature extraction. The complexity of this class derives from the number of equations used and segmentation, if applied.
*Digital Filter*	First derivative of ECG signal followed by digital filters followed by threshold [28].Bandpass filter applied to ECG signal followed by first derivative, followed by threshold [33].Bandpass filter applied before Hilbert transform, followed by threshold [39].Bandpass filter can be followed by first derivative before applying Wavelet transform, followed by threshold [38].Bandpass filter applied to ECG signal followed by matching filter (see Section 4.3), followed by threshold [40].	The digital filter can increase the SNR ratio depending on the nature of the filter and its order	The processed segments have equal and fixed lengths [23]–[28].The parameters used are fixed.The choice of differentiator in the digital filters functions as a notch filter.In the digital filter algorithms, the low-pass filter is usually a symmetrical amplification. The amplification values are determined experimentally.The mathematical operations (e.g. squaring, difference, multiplication) used are not justified by the authors.	The digital filters class of algorithms is simple and contains up to only four equations for feature extraction. The complexity of this class will increase if segmentation is applied. The order of complexity depends on the number of processed segments for each record.
*Mathematical Morphology*	Mathematical morphology filtering applied to ECG signal, followed by threshold [41].Mathematical morphology filtering applied to ECG signal, followed by first derivative, followed by threshold [34].	The signal noise is partially addressed by the mathematical morphology class of algorithms. The use of a low-pass filter improves the SNR.	The processed segments have equal and fixed lengths [23], [24], [25], [26], [27], [28].The structuring element is fixed during the ECG analysis.The length of the structuring element used is 3, which remains a fixed value.The length of the structuring element is determined experimentally. The length of the operating structure element must be shorter than the product of the length of the signal wave and the sampling frequency [41]. Therefore, the length of the structuring element can be different to 3.The authors do not justify the multiplication operations used [23], [24], [25], [26], [27], [28].	The mathematical morphology class of algorithms is simple and contains at least 15 equations for feature extraction. The complexity increases with the number of processed ECG segments. The order of complexity is higher than the derivative-based algorithms and digital filter algorithms.
*Empirical Mode Decomposition (EMD)*	EMD filtering applied to ECG signal followed by threshold [42].EMD filtering applied to ECG signal followed by singularity and threshold [43],[44].High-pass filter applied to ECG signal, followed by EMD filtering, followed by threshold [44].	The first several IMFs can filter out the noise and preserve the QRS content compared to the other ECG features [43].Therefore the first several IMFs are mainly caused by the QRS complex and improve the SNR.	The processed segments have equally fixed lengths [43].The number of IMFs depends on the length of the ECG segment. If the segment length is increased, the number of IMFs will increase.The length of the ECG segment is not determined experimentally.The choice of IMFs is determined by trial-and-error.	The EMD class of algorithms is simple and contains at least nine steps with several equations for feature extraction. The complexity increases with the number of processed ECG segments. Certainly, the order of complexity is higher than the derivative-based algorithms and digital filter algorithms.
*Hilbert Transform*	First derivative can be used before applying Hilbert transform followed by threshold [35], [36], [37].Bandpass filter applied before Hilbert transform, followed by threshold [39].Wavelet transform (WT), see Section 4.2, applied before Hilbert transform, followed by threshold [45].	The Hilbert transform does not improve the SNR itself. Therefore, some investigators filter the signal before applying the Hilbert transform. Benitez et al. [36] used a bandpass filter 8–20 Hz to remove muscular noise and maximise the QRS.	The processed segments have equally fixed lengths [36], [46].When the FFT approach was implemented in calculating the Hilbert transform, no dependence of the envelope on the frame width was detected for frames comprised of 512–2,048 data points.The length of the ECG segment is not determined experimentally.The choice digital filters and moving average are determined experimentally.	The Hilbert transform algorithm contains at least nine steps with several equations for features extraction. However, the primary disadvantage of this method is the increased computational burden required for FFT calculations compared to the time domain approaches. Hilbert transform techniques generally have a large computation overhead [46]. Moreover, the complexity increases with the number of processed ECG segments.
*Filter Banks*	Filter banks applied to ECG signal followed by threshold [47], [48].WT (see Section 4.2) applied to ECG signal, followed by filter banks, followed by correlation [49].	The filter banks significantly improve the SNR for Gaussian noise compared to the mean and median averaging methods [50]. For muscle noise, the filter banks improve the SNR more than the mean and median averaging methods [50].	The length of the filter, number of sub-bands, transition-band width and stop-band attenuation have fixed values [51]. For example, the length of each of the finite impulse response (FIR) filters used by Afonso et al. [50] was 32. The input noisy ECG is decomposed by the analysis filters into eight uniform sub-band frequencies. The sub-band signal in the (0–12.5 Hz) range is not modified. The sub-band signal in the (12.5–25 Hz) range is attenuated in the period outside the QRS complex. Any high-frequency components outside the QRS complex are modelled as noise. Thus, in the remaining six sub-bands (25–100 Hz*),* the signal is nulled in periods outside the QRS complex.The filter bank complexity depends on four parameters [51]: length of filter, number of sub- bands, transition-band width and stop-band attenuation. Theses parameters are determined experimentally.The main difficulty is choosing the optimal bank filters and their optimal combination in order to emphasise the QRS complexes.	The drawback of using filter banks is a relatively high computational cost due to the involvement of a large amount of multipliers in the FIR filters [48].
*Wavelet Transform (WT)*	WT applied to ECG signal, followed by threshold [52], [53].first derivative can be used before applying Wavelet transform followed by zero crossing (see section 5.6), followed by threshold [54].WT applied first before Hilbert transform, followed by threshold [55].WT applied to ECG signal, followed by filter banks, followed by correlation [56].WT applied to ECG signal, followed by neural networks (see Section 5.2) [54].Wavelet transform applied to ECG signal, followed by singularity (see 5.7) and zero crossing (see Section 5.6), followed by threshold [55].	WT does not increase the SNR, but the SNR can be improved by selecting the coefficients with the largest amplitude [56].	Choosing the mother wavelet is usually determined by the shape of the wavelet, which should be closer to the QRS complex shape, and it depends on the investigator's methodology for detecting the QRS complex.One mother wavelet (i.e. Haar, Daubechies, Biorthogonal, Mexican hat must be chosen once during the entire ECG analysis.Choosing the length of the processed ECG segment does vary in literature. Ahmed et al. [57] split the ECG signals into 2.4-seconds segments while Xiuyu et al. [55] split the signals into 11 seconds.Choosing the wavelet scale varies throughout the literature. Szilagyi and Szilagyi [58] used scales 2^3^ and 2^4^, which reflect the QRS complex, while Xu et al. [59] used scales from 2^2^ to 2^4^ to detect QRS complexes.In regards to the sampling frequency of the processed ECG signal, Martinez et al. [60] recommended to resample the signal at 250 Hz.	If the ECG is segmented (this is usually the case), the length of the segment reflects the tradeoff between accuracy and computational time-consumption of the algorithm [52]. In general, WT, similar to filter banks, is relatively high in computational cost [61].

### Amplitude

This algorithm is considered the oldest for detecting R peaks in ECG signals; however, for the last 30 years it is still useful and in common use. Recently, Sufi et al. [63] used the algorithm for detecting heart rate using mobile phone. In older algorithms, amplitude threshold was not used alone as in the case of Sufi et al. [63]; it was usually followed by a differentiation step to reduce the P and T wave influence relative to the R wave. The first derivative is applied after the amplitude threshold to accentuate the slope of the QRS complex. The amplitude threshold is calculated as a fraction of the measured ECG signal
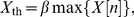
(1)where 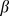 is the percentage of the ECG signal required to be removed and 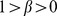. Different amplitude thresholds have also been used. Moriet-Mahoudeaux et al. [23] developed a QRS detector using 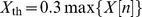, which means that 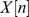 values below 30% of the maximum positive signal amplitude is truncated from the signal, while Fraden and Neuman [24] used 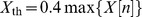.

### First Derivative Only

In this class of QRS enhancement algorithms, a first-order differentiator is commonly used as a high-pass filter, to enhance base-line wander and eliminate any undesired high frequency noise, modify the phase of the ECG signals, and to create zero crossings in the location of the R peaks. Many first derivative QRS detection algorithms, introduced in literature [31] calculate the first derivative of the measured ECG signal according to:
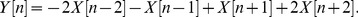
(2)In contrast, Holsinger [32] used a central finite-difference approach as:
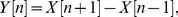
(3)whilst Okada [30] used a backward difference scheme:
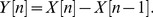
(4)


In these algorithms, a threshold criterion was subsequently applied to 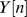 for QRS detection, as summarized in Table 1.

### First and Second Derivative

Note that, QRS enhancement algorithms compute the first and second derivatives of the measured ECG signal independently. A linear combination of the magnitudes of these derivatives then used to emphasise the QRS complex area relative to the other ECG features. In a seminal paper, Balda et al. [26] calculated the first and second derivatives of the measured ECG signals according to:
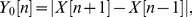
(5)

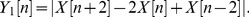
(6)


They then formed a linear combination of both derivatives as follows:
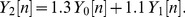
(7)


Ahlstrom and Tompkins [27] calculated the rectified first derivative of the ECG as:
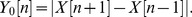
(8)


The rectified first derivative was then smoothed:
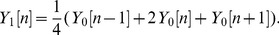
(9)


A rectified second derivative was then calculated:
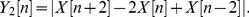
(10)


Finally, the rectified smoothed first derivative was added to the rectified second derivative:
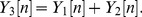
(11)


For all these algorithms, a threshold criterion for QRS detection was applied to the linear combination of derivatives. A summary of these threshold criteria is given in Table 1.

### Digital Filters

There have been many sophisticated digital filters for QRS enhancement published in the literature [28], [33], [64]–[71], as described briefly below. Algorithms utilizing more complex digital filters [28]–[30], [72]–[76] include Engelse and Zeelenberg [28], who first passed the ECG signal through a differentiator:
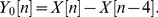
(12)


This signal was then passed through a digital low-pass filter:
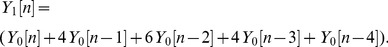
(13)


A different digital filter algorithm was introduced by Okada [30], who first smoothed using a three-point moving-average filter:
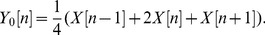
(14)


The output of this filter was then passed through a low-pass filter:
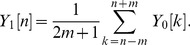
(15)


The difference between the input and output of this low-pass filter was then squared, in order to suppress low amplitude waves relative to the R peak:
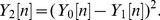
(16)


This square difference was then filtered, in order to enlarge the QRS area compared to the other ECG features:
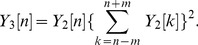
(17)


In addition to the above filters, a multiplication of backward difference (MOBD) algorithm has also been proposed [77], [78] for QRS detection. In brief, this approach consists of an AND-combination of adjacent magnitude values of the derivative. The MOBD of order 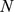 is defined by
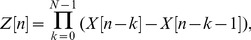
(18)where 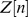 contains the extracted QRS features, which can subsequently be detected using an appropriate threshold. Another algorithm proposed by Dokur et al. [65] uses two different bandpass filters, subsequently multiplying the filter outputs 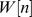 and 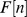 to form:
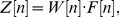
(19)where 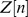 contains the extracted QRS features. This procedure is based on the assumption that each QRS complex is characterised by simultaneously occurring frequency components within the passbands of each filter. The multiplication operation performs the AND-combination. In other words, the output of the AND-combination (the feature output) is ‘true’, and therefore indicates a QRS complex, only if both filter outputs are ‘high’. The location of the maximum amplitude is taken as the location of the R wave. Conversely, Pan and Tompkins [33] used a derivative after applying a bandpass digital filter to the ECG signals. The bandpass filter consisted of a low-pass filter (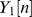) followed by a high-pass filter (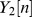) as:
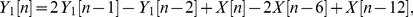
(20)

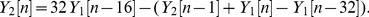
(21)


The first derivative (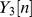) used after the bandpass filter was specified as:
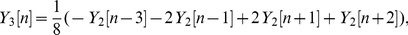
(22)


The bandpass filtered signal (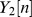) was differentiated to emphasise high signal slopes, suppressing smooth ECG waves and baseline wander.

### Mathematical Morphology

The use of mathematical morphology operators for QRS detection was described by Trahanias [79]. The mathematical morphology approach originates from image processing and was first proposed for ECG signal enhancement by Chu and Delp [80], who reported the successful removal of noise from the ECG using the approach. Mathematical morphology is based on the concept of *erosion* and *dilation*. Let 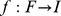 and 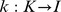 denote discrete functions, where the sets 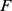 and 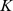 are given by 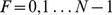 and 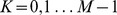. Here, 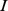 is the set of integer numbers. The *erosion* of the function 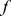 by the function 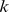 is defined as [80]:
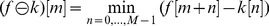
(23)where 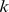 is also referred to as the *structuring element*, and 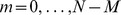. The values of 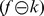 are always less than those of 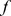. The *dilation* of the function 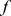 by the function 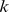 is defined as [80]

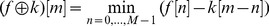
(24)where in this case 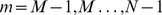. The 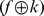 values of are always greater than those of 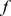. Erosion and dilation may be combined for additional operations. *Opening*, denoted by 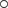, is defined as erosion followed by dilation. *Closing*, denoted by 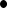, is defined as dilation followed by erosion. Both operators manipulate signals in a comparable way. That is, to *open* a sequence 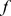 with a flat structuring element 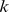 will remove all peaks. To *close* the sequence with the same structuring element will remove all pits (negative peaks). In Trahanias [79], opening and closing operations are used for noise suppression as proposed by Chu and Delp [80]; that is:
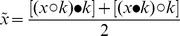
(25)where 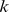 is a flat structuring element (zero line). The generation of a feature signal for the QRS complexes is accomplished by the operation
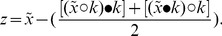
(26)Zhang and Lian [34] used the first derivative after multiscale mathematical morphology filtering to the ECG signal in order to remove motion artifacts and base line drifts. They used Okada's first-order differential equation, as shown in Equation 4.

### Empirical Mode Decomposition

Empirical mode decomposition (EMD) was introduced by Huang et al. [81] for nonlinear and non-stationary signal analysis. The key part of this method is that any complex data set can be decomposed into a finite and often small number of intrinsic mode functions (IMFs), which admit well-behaved Hilbert transforms. Usually, when the raw ECG signals are decomposed into number of IMFs, the combination of IMFs produces a resulting signal where the QRS complex is more pronounced. This process can be considered as adaptive filtering, similar to the use of wavelet transform. The EMD is defined by a process called sifting. It decomposes a given signal into a set of components, the IMFs. 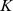 modes 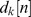 and a residual term 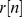 [82], [83] are obtained and expressed by:
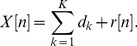
(27)


The EMD algorithm is summarised by the following steps:

1. Start with the signal 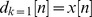; followed by the sifting process 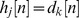, 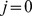.
2. Identify all local extrema of 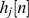.
3. Compute the upper (EnvMax) and lower envelopes (EnvMin) by cubic spline interpolation of the maxima and minima.
4. Calculate the mean of the lower and upper envelopes, 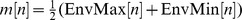.
5. Extract the detail 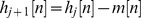.
6. If 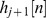 is an IMF, go to step 7; otherwise, iterate steps 2 to 5 on the signal 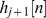, 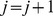. (The definition of an IMF, although somewhat vague, consists of two parts: (a) the number of the extrema equals the number of zeros and (b) the upper and lower envelopes should have the same bsolute value).
7. Extract the mode 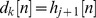.
8. Calculate the residual 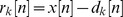.
9. If 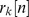 has less than two extrema, the extraction is finished 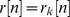; otherwise, iterate the algorithm from step 1 on the residual 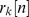, 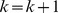.

### Hilbert Transform

The use of the Hilbert transform for QRS detection is proposed by Zhou et al. [84] and Nygards and Srnmo [85]. In the time domain, the Hilbert transform of the ECG signal 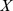 is defined as:
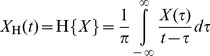
(28)

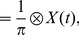
(29)where 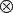 denotes the convolution operator. In the frequency domain, the ECG signal can be transformed with a filter of response:
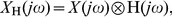
(30)where the transfer function of the Hilbert transform 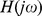 is given by:
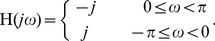
(31)


Using the numerically efficient Fast Fourier Transform (FFT), the Hilbert transform can easily be computed. The Hilbert transform 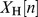 of the ECG signal 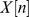 is used for the computation of the signal envelope [85], which is given for band-limited signals by
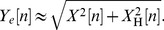
(32)


A computationally less expensive approximation to the envelope can be made by [85]

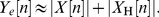
(33)


To remove ripples from the envelope and to avoid ambiguities in the peak level detection, the envelope is low-pass filtered in Nygards and Srnmo [85]. Additionally, they propose a waveform adaptive scheme for the removal of low-frequency ECG components is proposed. The method of Zhou et al. [84] is related to the algorithms based on the Hilbert transform. In their study, the envelope of the signal is approximated using
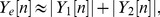
(34)where and are the outputs of two orthogonal digital filters, namely:
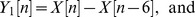
(35)

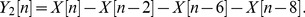
(36)


In order to remove noise, the envelope signal 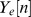 is smoothed by a four-tap moving average filter. Some investigators use a first derivative before applying the Hilbert transform [35]–[37]. Differentiating the ECG modifies its phase, creating zero crossings at the presumed location of the R peaks. Thus, a transformation is required to rectify the phase in order to create a signal with marked peaks at the true location of the R peaks.

### Filter Banks

Filter banks decompose the bandwidth of the input ECG signal into sub-band signals with uniform frequency bands. The sub-bands can be downsampled, since the sub-band bandwidth is much lower than the input signal. The sub-bands provide information from various frequency ranges; thus, it is possible to perform time- and frequency-dependent processing of the input signal.

**Figure 3:**
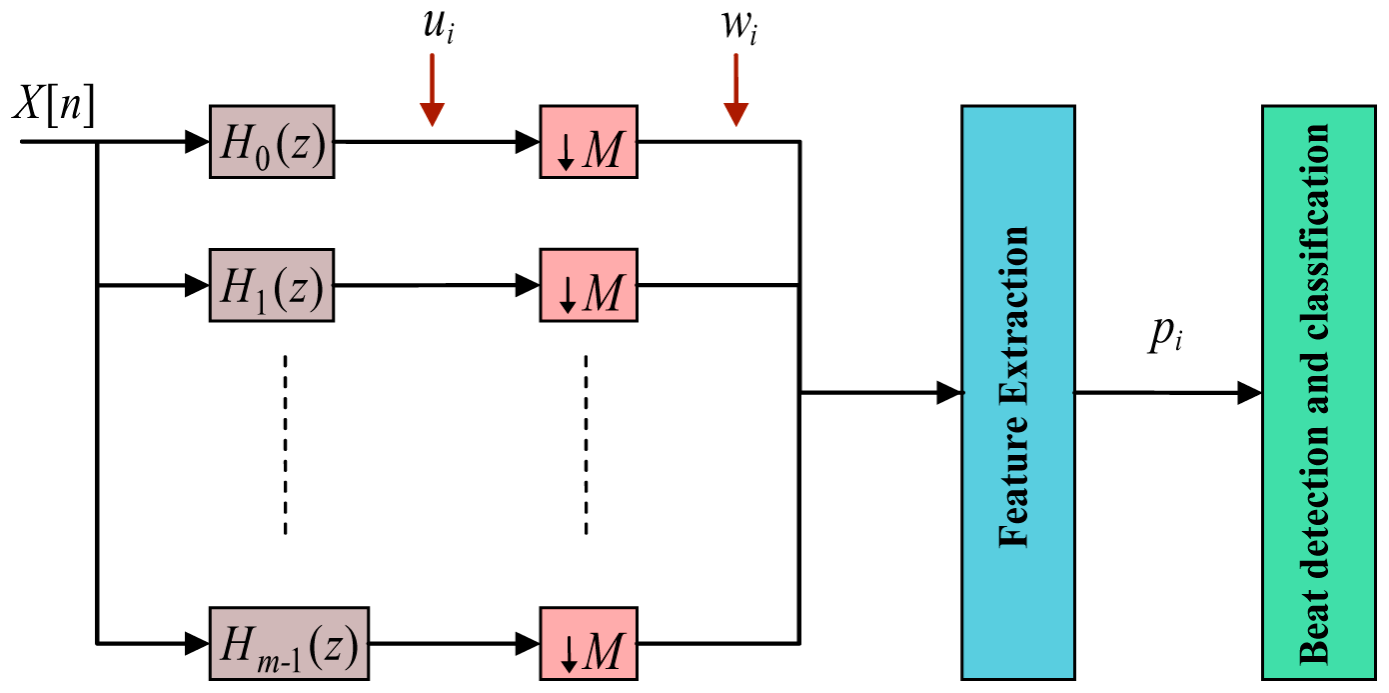
Filter bank schematic. A filter bank contains a set of analysis filters that decompose the input signal into sub-bands 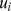 with uniform bandwidths in order to extract ECG features. Here, 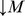 is a downsampling process producing down-sampled signals 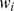.

As shown in Figure 3, a filter bank contains analysis filters, which decompose the input signal into sub-band signals with uniform frequency bandwidths, each of constant length. The analysis filters bandpass the input ECG signal to produce the sub-band signals:
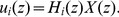
(37)


The effective bandwidth of 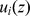 is 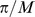 and 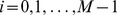; thus, they can be downsampled to reduce the total rate. The downsampling process 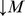 (Fig. 3), keeps one sample out of all samples. The downsampled signal 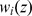 is
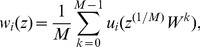
(38)where 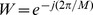. The sub-bands 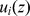 and 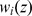 are bandpassed versions of the input, and 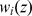 has a lower sample rate than 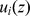. The filtering process can be efficiently conducted at 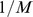 the input rate by taking advantage of the downsampling. This process is referred to as polyphase implementation and it contributes to the computational efficiency of filter bank algorithms [47]. A variety of features indicative of the QRS complex can be designed by combining sub-bands of interest reported in Afonso et al. [47]. For example, a sum-of-absolute values feature can be computed using sub-bands, 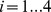. From these sub-bands six features (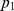, 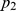, 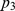, 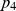, 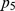, and 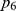) can be derived as follows:
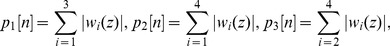
(39)

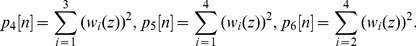
(40)These features have values that are proportional to the energy of the QRS complex. Finally, heuristic beat-detection logic can be used to incorporate some of the above features that are indicative of the QRS complex.

### Wavelet Transform

Wavelets are closely related to filter banks. The wavelet transform (WT) [86] of a function 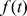 is an integral transform defined by
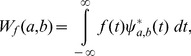
(41)where 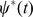 denotes the complex conjugate of the wavelet function 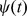. The transform yields a *time-scale* representation similar to the *time-frequency* representation of the short-time Fourier transform (STFT). In contrast to the STFT, the WT uses a set of analysing functions that allow a variable time and frequency resolution for different frequency bands. The set of analysing functions—the wavelet family 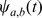
—is deduced from a *mother wavelet* 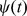 by:
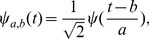
(42)where 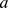 and 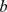 are the *dilation* (scale) and *translation* parameters respectively. The scale parameter a of the WT is comparable to the frequency parameter of the STFT. The mother wavelet is a short oscillation with zero mean. The discrete wavelet transform (DWT) results from discretised scale and translation parameters; for example, 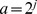 and 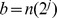, where 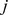 and 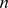 are integers. This choice of 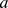 and 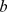 leads to the dyadic WT (DyWT):
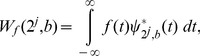
(43)

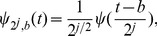
(44)

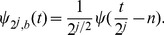
(45)


Although defined as an integral transform, the DyWT is usually implemented using a dyadic filter bank where the filter coefficients are directly derived from the wavelet function used in the analysis [87]–[89].

## QRS Detection

After enhancing the QRS features using the previous algorithms, the next step is to detect the QRS complexes. Through the previous enhancement step, QRS complexes are filtered and magnified relative to other ECG features and noise. There are many detection techniques used in the literature, as shown in Table 2. This include thresholding, neural networks [91], [112]–[114], hidden Markov model [95], matched filters [115], [116], syntactic methods [104]–[106], zero-crossing [107], and singularity techniques [117]–[119]. In the summary of Table 3, all these algorithms are numerically inefficient except thresholding. As the main purpose of this article is to highlight suitable algorithms for ECG monitoring using battery-operated, portable devices, only thresholding will be considered for the detection phase for simplicity and efficiency. In this context, it has to be emphasised that thresholding can be applied to time-domain [23], [24], [120] as well as time-frequency [121]–[123] ECG signals. However, the use of a fixed threshold to detect QRS complexes is simple and only efficient for stationary ECG signals with similar beat-to-beat morphology. Due to severe baseline drift and movement of patients, an ECG waveform may vary drastically from one heartbeat to the next in mobile applications. Therefore, the probability of not accurately detecting QRS complexes is high. Using adaptive thresholding [59], [124]–[126], the probability of missing QRS complexes decreases. However, the main drawback of these adaptive-thresholding based algorithms is the setting of multiple thresholds empirically. Therefore, currently, these algorithms cannot provide a universal solution to the QRS detection problem, since they may work perfectly on some clean signals, but not those containing arrhythmias or noisy QRS complexes.

**Table 2:** Comparison of QRS detection techniques based on algorithm usage and assessment criteria.

Technique	Algorithm	Robustness to noise	Parameter choice	Numerical efficiency
*Threshold*	The threshold step has been used in the literature as the last stage for most QRS detection algorithms [23], [24], [26], [27], [30], [31], [32], [34], [35], [36], [37], [38].	The performance of the threshold approach will be affected by low SNR signals [29], [33].	–The threshold is a fixed value [26], [28], [31], [33].–The threshold is experimentally defined [26], [28], [31], [33]. The real difficulty is in choosing the optimal threshold.	The threshold approach is simple. It is an IF-THEN-ELSE statement. Therefore, it is considered computationally efficient by researchers [26], [28], [31], [33].
*Neural Networks (NN)*	–WT applied to ECG signal, followed by NNs [54]– Wavelet applied first to ECG signal, followed by Hidden Markov Model [90].– NNs (used as a filter) applied to ECG signal, followed by a matched filter [91].	NN are highly sensitive to noise [92]. The performance of the classifier can be significantly reduced if the NN is constructed with a proper architecture and trained with appropriate data.	– The type of the NNs must be chosen and adjusted before the analysis.–Number *N* of inputs to NNs: to have just one single NN with a fixed number *N* of inputs, with each one receiving one of the samples from the window. The number of samples per window must then be fixed [93].– There is a range of samples to be selected as the number of NN inputs, for example, García-Berdonés et al. [93] used 20 samples as the number of inputs.– Choosing the number of neurons in the NN hidden layer still remains a challenge. There is no definite way of determining the right number of neurons in hidden layer.	–The training phase can be numerically inefficient as it is an iterative process for adjusting the NN weights [94]. If the number of hidden neurons is large, the computational load for training is high.– Even while the NN is implemented only in the trained version on the mobile device, it often needs a considerable amount of memory to store the neuron weights. Moreover, a nonlinear (most often sigmoid) function needs to be evaluated in the operating phase, which is computationally inefficient.
*Hidden Markov Models (HMM)*	–Bandpass filter applied to ECG signal, followed by HMM [95], [96].–Wavelet applied to ECG signal, followed by HMM [90].	–HMM is sensitive to noise, baseline wander and heart rate variation [97].	– Determining the number of states, transition probabilities and output function has been done experimentally.–The parameters of a HMM cannot be directly estimated from training data using maximum likelihood estimation formulas, since the underlying state sequence that produced the data is unknown [95].–HMM parameters are to be fixed.	–The problems of the method include a necessary manual segmentation for training prior to the analysis of a record, its patient dependence, and its considerable computational complexity, even when the computationally efficient Viterbi algorithm [98] is applied.–The number of parameters that need to be set in a HMM is large-there are usually 15 to 50 parameters that need to be evaluated [95], [96].
*Matched Filters*	– Matched filters applied to ECG signal [99].– Digital filter applied to ECG signal, followed by matched filters [100], [101].–NNs (used as a filter) applied to ECG signal, followed matched Filter [91].	The matched filter improves SNR [102].	– Fixed template length.–The template length and filter are determined experimentally.	Efficient implementations are available [103]. In general, however, it is computationally expensive because of the sample-by-sample moving comparison with the template along the ECG signals.
*Syntactic Method*	The syntactic method is applied to an ECG signal to detect a QRS complex by itself [104]–[106].	The syntactic method is sensitive to noise [106].	–The length of the segment is fixed. Belforte et al. [104] used 30-seconds duration per segment.– Four fixed attributes used the syntactic method [105]: degree of curvature, arc length, chord length and arc symmetry, which are determined experimentally.	The syntactic method has a high computational cost compared to other approaches. Measurements of various parameters have to be performed; powerful grammars capable of describing syntax as well as semantics are needed to model the formulation of a pattern grammar.
*Zero- Crossing*	The zero-crossing technique has been used in the literature to detect QRS complexes as follows:– Bandpass filter applied to ECG signal, followed by zero crossing [107].– WT applied to ECG signal, followed by zero crossing, followed by threshold [107].– WT applied to ECG signal, followed by singularity and zero crossing, followed by threshold [55].	The zero crossing is sensitive to noise [107].	–The threshold used for counting the number of zero crossings per segment is fixed [107] and determined experimentally.–Choosing the wavelet scales to search for zero-crossing varies in literature [107], [108].	The zero-crossing approach is simple but computationally inefficient. This is because of the time consuming stages in the maximum/minimum search for temporal localization of the R wave [107].
*Singularity*	–EMD filtering applied to ECG signal, followed by singularity and threshold [43].– WT applied to ECG signal followed by singularity and zero crossing, followed by threshold [55].	The singularity approach is sensitive to noise [109].	–Choosing the wavelet scales to search for singular points is performed experimentally [109], [110].–The threshold used for detecting R peaks per segment is fixed [109].–The threshold used for detecting R peak counts per segment is determined experimentally.	The singularity approach load is more complex than the zero- crossing approach. It is computationally inefficient because of the consuming stages in the search and optimization for detecting R waves in ECG segments [55], [109].

**Table 3:** Comparison of ECG beat detection algorithms based on techniques for QRS enhancement and detection on the MIT-BIH arrhythmia database [62].

Publication	QRS Enhancement	QRS detection	Number of beats	Numerical Efficiency	SE (%)	+P (%)
Chiarugi et al. [136]	Bandpass Filter + first Derivative	Multiple thresholds	109494	High	99.76	99.81
Christov [124]	Multiple moving averages + first derivative	Multiple thresholds	109494	High	99.76	99.81
Elgendi [137]	Bandpass filter + first derivative + squaring	Thresholding using two moving averages	109985	High	99.78	99.87
Zidelmal [138]	WT + coefficients multiplication	Two thresholds	109494	Medium	99.64	99.82
Choukri [139]	WT + histogram +moving average	Two thresholds	109488	Low	98.68	97.24
Li et al. [127]	WT + digital filter	Singularity + multiple thresholds	104182	Low	98.89	99.94
Pan and Tompkins [33]	Bandpass filter+first derivative + squaring + moving average	Multiple thresholds	116137	Medium	99.76	99.56
Arzeno et al. [35] and Benitez et al. [36]	First derivative + Hilbert transform	Threshold	109257	Medium	99.13	99.31
Arzeno et al. [35]	First derivative + Hilbert transform	Two thresholds	109517	Medium	99.29	99.24
Arzeno et al. [35]	First derivative + squaring + bandpass filter	Multiple thresholds	109504	Medium	99.68	99.63
Arzeno et al. [35]	First derivative + squaring + bandpass filter	Variable thresholds comparison	109436	Medium	99.57	99.58
Arzeno et al. [35]	Second derivative + squaring + bandpass filter	Variable thresholds comparison	108228	Medium	98.08	99.18
Moraes et al. [110]	Low pass filter + First derivative + modified spatial velocity	Threshold	109481	Medium	99.69	99.88
Chouhan and Mehta [111]	Digital filters	Threshold	102654	Medium	99.55	99.49
Elgendi et al. [125]	Digital filters	Multiple thresholds	44677	Medium	97.5	99.9
Martinez et al. [60]	WT	Multiple thresholds + zero Crossing	109428	Medium	99.8	99.86
Afonso et al. [141]	Filter banks	Multiple thresholds	90909	Low	99.59	99.56
Ghaffari et al. [121]	Continuous WT	Threshold	109837	Medium	99.91	99.72
Zheng and Wu [122]	Discrete WT + Cubic Spline Interpolation + moving average	Threshold	N/R	Low	98.68	99.59
Ghaffari et al. [121]	Hybrid Complex WT	Threshold	24000	Low	99.79	99.89
Ghaffari et al. [121]	Complex Frequency B-Spline WT	Threshold	24000	Low	99.29	99.89
Ghaffari et al. [121]	Complex Morlet WT	Threshold	24000	Medium	99.49	99.29

SE and +P stand for sensitivity and positive productivity respectively, while N/R denotes not reported.

## Discussion

The performance of QRS detection algorithms are typically assessed using two statistical measures: sensitivity 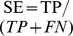 and positive predictivity 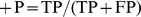, where 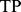 is the number of true positives (QRS complexes detected as QRS complexes), 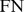 is the number of false negatives (QRS complexes which have not been detected), and 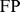 is the number of false positives (non-QRS complexes detected as QRS complexes). The sensitivity reports the percentage of true beats that were correctly detected by the algorithm, whilst the positive predictivity reports the percentage of beat detections that were true beats.

The performance of current QRS detection algorithms described in the literature has not been completely assessed in terms of robustness to noise, parameter choice, and numerical efficiency. Moreover, many of the QRS algorithms have not been tested against a standard database, or any database at all making the results difficult to compare and evaluate. Furthermore, many algorithms scored a high detection performance using the overall number of detected beats (i.e. QRS complexes), as shown in Table 3. It is worth noting that the algorithm of Li et al. [127] scored high overall performance with a sensitivity of 99.89% and a specificity of 99.94%. However, Li et al. excluded files 214 and 215 from the MIT-BIH arrhythmia database [62], and therefore their algorithm may not superior in terms of performance. In addition, their algorithm was based on wavelet feature extraction and singularity for classification, which is considered numerically inefficient.

As noted, some investigators have excluded records from the MIT-BIH arrhythmia database [62] for the sake of reducing noise in the processed ECG signals; consequently their algorithms appeared to achieve improved performance. Other researchers excluded segments with ventricular flutter [60] and signals from patients with paced beats [110] from their investigations. Therefore, a robust algorithm is required to analyse ECG signals without excluding any records or particular segments, especially if the main goal is to provide a robust algorithm for long-term ECG signals recorded over a few days.

### Robustness to Noise

Robustness to noise is effectively tested as we use signals from the widely used MIT-BIH Arrhythmia Database [62] that contains signals with different noise sources and non-sinus beats. The MIT-BIH database is widely used to evaluate QRS detection algorithms. As demonstrated in Table 1, there are many algorithms used for denoising and enhancing the QRS complex in ECG signals.

Usually, denoising ECG signal requires a bandpass filter, which can be implemented on battery-driven devices and while reasonably preserving the clinical features of ECG signals (P, QRS, and T waves) at the same time. Perhaps, a more sophisticated algorithm may filter the ECG more effectively, for example Sameni et al. [128] proposed a Bayesian framework that filters ECG better than the conventional bandpass filtering [129]–[131], adaptive filtering [132], and wavelet denoising [133], [134] over different types of noise using highly realistic synthetic ECG. Recently, Sharma et al. [135] proposed a wavelet-based denoising method tested on real ECG data and synthetic ECG signals. However, both algorithms are numerically inefficient.

Table 3 shows that the Chiarugi et al. [136] as well as Christov [124], and Elgendi [137] algorithms are highly-numerically efficient, and the use of a first derivative with or without moving average in the QRS enhancement phase is promising, especially when it is followed by a proper QRS detection phase such as moving average and/or dynamic threshold. However, the only use of derivative in the QRS enhancement phase without a proper QRS detection phase is extremely sensitive to noise [29].

It is worth noting that Elgendi's algorithm [137] tested on the MIT-BIH Noise Stress Test Database and scored higher accuracy in detecting R peaks compared to Pan-Tompkins [33] and Benitez et al. [36].

### Battery-Driven ECG Devices

Many QRS detection algorithms have been published, and a comparison between them needs to be conducted. An algorithmic comparison regarding numerical efficiency has been carried out empirically. As shown in Table 3, each algorithm has been categorised as low, medium or high in terms of its numerical efficiency, based on the number of iterations and the number of equations (e.g. multiplications, additions, differentiations) employed. The better the numerical efficiency, the faster the algorithm, and vice-versa. Consequently, the faster the algorithm, the more suitable it is for real-time monitoring.

With advances in computational power, the demand for numerical efficiency has decreased. However, this is still more the case when the ECG signals are collected and analysed in hospitals, but not for the case of portable ECG devices, which are battery driven. This leads to especially high demands on algorithms for use within a mobile phone for monitoring ECG signals of patients in a mobile, unobtrusive at home setting. Therefore, there is a need for developing numerically efficient algorithms to accommodate the new trend towards mobile ECG devices and to analyse long-term recorded signals in a time-efficient manner.

Typically, processing large databases is carried out on PC workstations with high-speed, multi-core processors and efficient memory. This advantage is still not available for battery-operated devices: even the current smartphone platforms have limited RAM and processing power [8], [10], [142]. In general, battery-driven ECG devices follow one of these schemes: 1) collect data for offline analysis; 2) collect data for real-time analysis within the device itself; or 3) collect data for real-time analysis via a remote connection to a separate server. Certainly, each scheme has its own advantage and disadvantage in terms of processing time and power consumption.

The Holter device is the most commonly-used ECG battery-operated platform, especially for monitoring and recording ECG signals to be processed offline. With the advancement of smartphones in terms of memory and processors, investigators are trying to replace the Holter devices by smartphones [8]. Furthermore, the use of a smartphone has extra advantages from the patient perspective such as mobility, familiarity and guaranteed usage [143]. Thus, recently, there have been some contributions in phone applications that analyse ECG signals collected wirelessly via Bluetooth [8], [10], [144] and Zigbee radio protocols [142].

**Figure 4:**
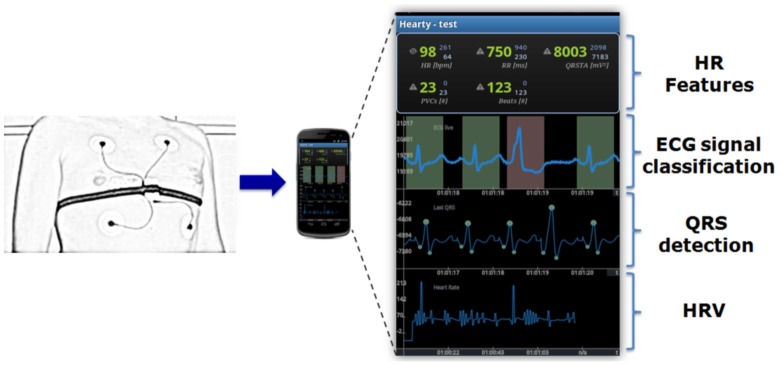
Screenshot showing the main interface of the ‘Hearty’ application implemented by Gradl et al. (2012) [8]. From top to bottom: Panel showing various clinically relevant parameters that are automatically detected including heart rate (HR) and RR interval; Panel showing the detected ECG signal, which is wirelessly streamed to the application; Panel showing the QRS detection with filled circle markers for the Q, R and S waves; Panel showing the detected beat-to-beat heart rate.

The current advances in battery-driven devices such as smartphones and tablet computers have made these technologies invariably part of daily life, even in developing countries [12]. It has also increased the possibility of implementing more sophisticated algorithms such as the Pan-Tompkins method [33] on smartphones as shown in Figure 4. However, there is a significant trade-off as there will always be a power-consumption limitation in processing ECG signals on battery-operated devices. Therefore, prior to deploying any algorithm on modern mobile devices, comprehensive evaluation of the algorithm based on robustness to noise, parameter choice, and numerical efficiency is required to improve the quality of diagnosis with respect to processing time or power consumption. One of the recent studies that confirms this recommendation is done by Hyejung et al. [145] who developed a simple algorithm to detect QRS complexes for Holter devices. Their simple algorithm, which consists of bandpass filter followed by multiple thresholds, was faster and more efficient compared to relatively more complex methods [35], [146].

### Mobile Telemedicine Systems

Mobile telemedicine systems often use mobile phones/PDAs to just collect the ECG data–wirelessly or wired–and send them to a central monitoring station using GSM or internet for further analysis [147], [148]. In such cases, some analysis can be done locally on the smartphone before transmission; however, it is not always recommended as the transmission can consume more power than the ECG analysis itself [149]. There is no doubt that the essential quality for any algorithm used for real-time analysis is its simplicity (numerical efficiency), provided this does not decrease accuracy. The simpler the algorithm (while retaining accuracy), the faster it will be in processing large databases [35], [150], and it will consume less power for battery-operated devices [63], [142], [151]. Moreover, a simple algorithm also offers low cost of hardware implementation in both power and size for body sensor networks [152]. Sufi et al. [163] investigated three simple QRS algorithms suitable for mobile phones. The QRS enhancement phase of these algorithms consisted of amplitude, first-derivative, and second-derivative methods, while the QRS detection phase was threshold-based. They used simple methodologies for QRS enhancement and detection for implementation over mobile phones. This simplicity has been confirmed in Table 3 where it is evident that the first derivative and threshold are an efficient combination for detecting QRS if developed properly.

### Mobile Phone Applications

**Figure 5:**
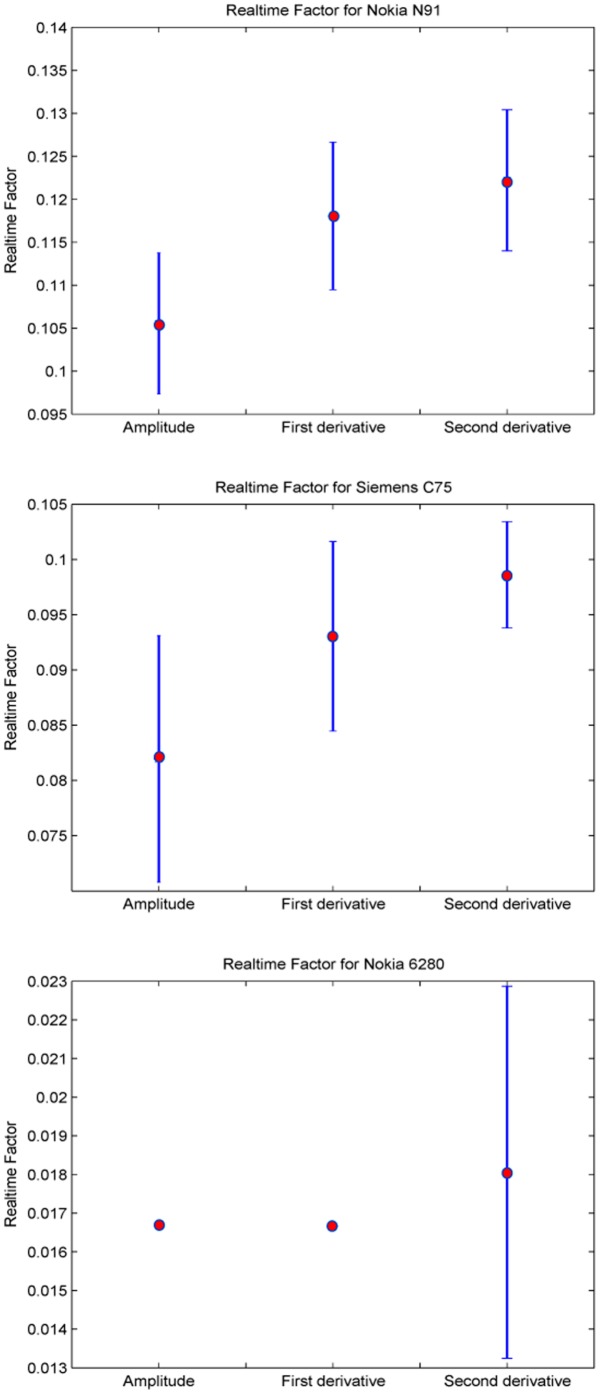
A showcase of realtime factors for three outdated mobile phones. Three QRS detection algorithms were tested, as reported by Sufi et al. [63]. The QRS enhancement phase was based on amplitude, first-derivative, and second-derivative techniques, whilst the QRS detection phase employed thresholding. Realtime factor is the processing time needed to run the QRS detection algorithm for an individual ECG entry within one measurement window size of 60 seconds.

**Figure 6:**
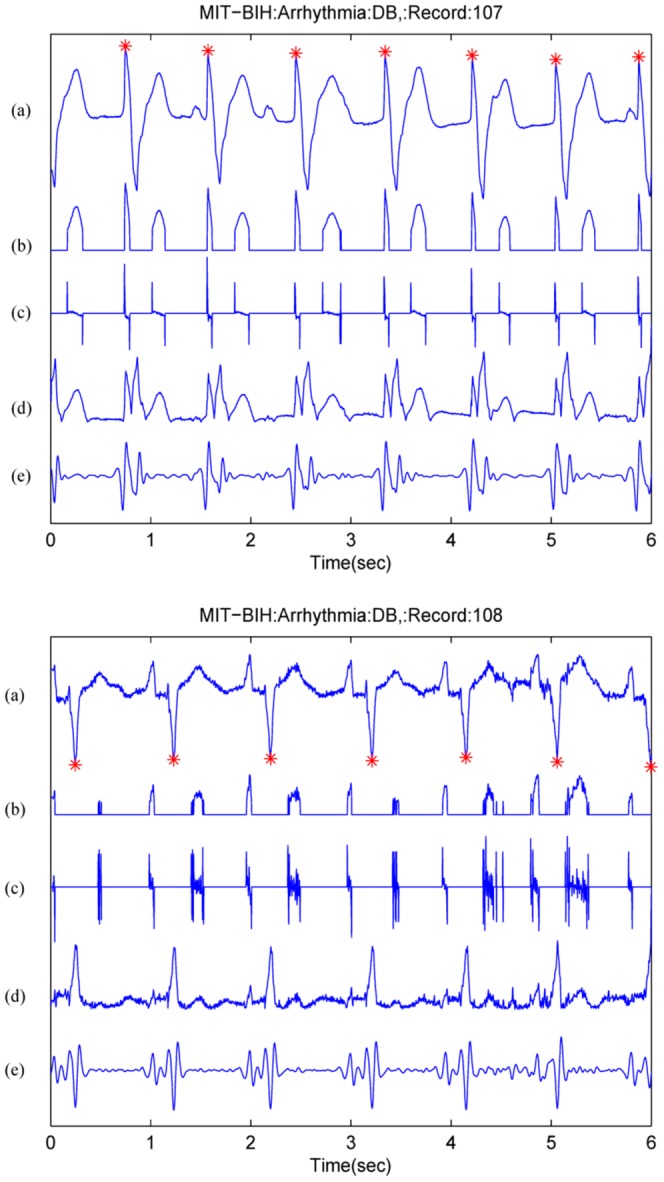
QRS enhancement stage in ECG signals. (a) ECG signal (top: from record 107, bottom: from record 108 of the MIT-BIH Arrhythmia Database [62]), (b) amplitude from Eq.1 where 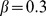, (c) first derivative from Eq.4, (d) first derivative and second derivative from Eq.7, and (e) digital filter from Ref. [33]. Signal amplitudes have been manipulated to fit all signals in one figure. Here, a red asterisk represents the annotated R peak.

To demonstrate the importance of processing time on a mobile phone, a showcase is demonstrated in Figure 5 for three outdated mobile phones [63]. It can be seen that the Nokia 6280 consumes the least processing time, as shown in Fig. 5(c). As expected, the amplitude-based QRS enhancement technique was faster than the first-derivative and second derivative based techniques. In this study [63], the quality of ECG signals was discussed and the data used was relatively noise-free, as the ECG signal shown in Figure 2 illustrates. However, this does not mean that a simple (or faster) algorithm will be more accurate. For example, Figure 6 shows that a simple amplitude threshold or first derivative method does not emphasize the QRS complex for the case of paced beats (record 107) and inverted QRS complexes (record 108). Nevertheless, the Sufi et al. result is considered a foundational step for monitoring ECG signals using mobile phones, but their algorithm exhibited some limitations in terms of memory and processing time.

Nowadays, smartphones possess advanced processing and storage capabilities, including a powerful CPU, more memory and GPUs with high-speed data access via Wi-Fi or mobile broadband [153]. Therefore, implementing a sophisticated QRS detection algorithm on a smartphone is becoming more feasible. Gradl et al. [8] implemented the Pan-Tompkins algorithm [33] on three smartphones: Samsung GT-I9000, Samsung™ GT-N7000, and HTC™ Wildfire S A510e. The authors showed that processing of the wirelessly streamed ECG signal is feasible in real-time on the mentioned devices; however, they stated that battery lifetime was affected negatively by running the monitoring application.

Certainly, the implementation of the Pan-Tompkins algorithm is more resource-demanding, and therefore consumes more time and power, than the three simple algorithms investigated by Sufi et al. [63]. Nevertheless, recent wearable devices can easily fulfil the real-time requirement. For example, the real-time factor for processing record 100 of the MIT-BIH Arrhythmia Database [62] using the Pan-Tompkins algorithm over three recent tablet computers/smartphones: the Asus Transformer Prime, the Samsung Galaxy S III, as well as the Samsung Galaxy S II was 0.14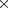, 0.13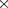, and 0.2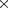, respectively. In contrast, the real-time factors for processing the same record using the first-derivative algorithm on the outdated phones: the Nokia N91, the Siemens C75, and the Nokia 6280 were 0.13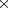, 0.1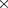, 0.016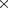, respectively.

Another aspect that has been ignored in the literature is the clinical utility of the ECG algorithms. It is rare to find a study that addresses the usefulness of the developed algorithm in a clinical setting. As far as we are aware, there is no evidence that shows whether the discussed algorithms are currently implemented and tested in clinical settings.

## Conclusions

In conclusion, we provide a summary of the required algorithms for ECG detection based on the literature together with our own investigations. The use of the first-derivative of the filtered ECG with or without a moving-average filter is recommended, as this approach is highly numerically efficient for the QRS enhancement phase, but is sensitive to noise and arrhythmia; therefore, an adaptive thresholding or integration-based approach is needed in the detection phase. Both of these suggested methodologies are simple and computationally efficient for the detection of QRS complexes in mobile-phone applications. If more processing power is available, as is the case on modern tablet computers and smartphones, implementation of the classical Pan-Tompkins algorithm [33] is also a feasible choice. Overall, simplicity and efficiency are required in developing QRS detection algorithms for processing long-term recordings and large databases, as well as for expanding our telemedicine capabilities in the near future.
